# Alpha subunit-dependent glycine receptor clustering and regulation of synaptic receptor numbers

**DOI:** 10.1038/s41598-017-11264-3

**Published:** 2017-09-07

**Authors:** A. Patrizio, M. Renner, R. Pizzarelli, A. Triller, C. G. Specht

**Affiliations:** 1 École Normale Supérieure, PSL Research University, CNRS, Inserm, Institute of Biology (IBENS), Paris, 75005 France; 2 Université Pierre et Marie Curie, Institut du Fer à Moulin (IFM), Paris, 75005 France

## Abstract

Accumulation of glycine receptors at synapses requires the interaction between the beta subunit of the receptor and the scaffold protein gephyrin. Here, we questioned whether different alpha subunits could modulate the receptors’ diffusion and propensity to cluster at spinal cord synapses. Using quantitative photoactivated localisation microscopy we found that alpha-1 and alpha-3 containing glycine receptors display the same α_3_:β_2_ stoichiometry and gephyrin binding. Despite these similarities, alpha-3 containing receptors are less mobile and cluster at higher density compared to alpha-1, with 1500 versus 1100 complexes µm^−2^, respectively. Furthermore, we identified a subunit-specific regulation of glycine receptor copy numbers at synapses: when challenged with interleukin 1β, the synaptic occupancy of alpha-1 but not alpha-3 receptors was reduced. This mechanism may play a role in the cell-type dependent regulation of glycinergic currents in response to interleukin 1β and highlights the capacity of the alpha subunits to affect receptor-gephyrin binding at synapses.

## Introduction

The clustering of inhibitory neurotransmitter receptors at synapses is largely dependent upon their interaction with the scaffold protein gephyrin. In the case of the glycine receptor (GlyR), high affinity binding is conferred by sequences in the large intracellular loop of the β subunit of the receptor^1^. GABA_A_ receptor (GABA_A_R) subunits compete for the same receptor binding sites, located in the C-terminal E domain of gephyrin^2^. The strength of receptor-gephyrin interactions determines their dynamic equilibrium and controls receptor accumulation at synapses. Post-translational modifications that have an impact on the strength of receptor-gephyrin binding thus induce plastic changes in the number of receptors at synapses^3–6^.

GlyRs can bind to gephyrin outside of synapses, too, in line with the role of gephyrin in the forward trafficking of GlyRs^7^. As shown by single particle tracking, gephyrin binding reduces the speed of diffusion of GlyRs in extrasynaptic regions of the plasma membrane, suggesting that a substantial proportion of receptors are bound to gephyrin^8^. Hence, the accumulation of GlyRs at synapses is dependent also on gephyrin-gephyrin interactions that regulate the synaptic clustering of the scaffold protein^9^. Post-translational modifications that alter the strength of gephyrin-gephyrin binding modulate gephyrin and GlyR numbers at synapses alike (e.g.^10^).

These findings imply that in heteromeric GlyRs composed of α and β subunits, the α subunits do not contribute to the clustering at synapses, but rather control the functional properties of the receptor. After all, the α subunits are required for ligand-binding and allosteric modulation^11, 12^ and are the target of signalling processes that regulate GlyR function. PKA-dependent phosphorylation of the GlyRα3 subunit was shown to reduce glycinergic inhibition in dorsal horn interneurons in the context of inflammatory pain^13^. Similarly, the pro-inflammatory cytokine interleukin 1β (IL-1β) causes rapid changes in glycinergic currents in different populations of spinal cord interneurons^14, 15^.

In contrast to this view we hypothesise that the functional and dynamic properties of GlyRs are not strictly separated between the α and the β subunits of the receptor. Such is the case for the GABA_A_R, where receptor diffusion is regulated in response to agonist binding and allosteric modulation^16^. To determine a possible role of the α subunit in GlyR clustering we compared the assembly of α1 and α3 containing GlyRs and their accumulation at synapses using a single molecule counting approach based on photoactivated localisation microscopy (PALM). We observed α subunit-dependent clustering of GlyRs in spinal cord neurons, and identified the α1 specific displacement of GlyRs in response to IL-1β. Our data reveal a molecular mechanism that controls GlyR clustering by acting upon the α subunits of the receptor. The differential regulation of α1 and α3 containing GlyRs may underlie neuron-specific changes of glycinergic inhibition during inflammatory processes.

## Results

### α-subunit specific diffusion of GlyRs at synapses

**Figure 1:**
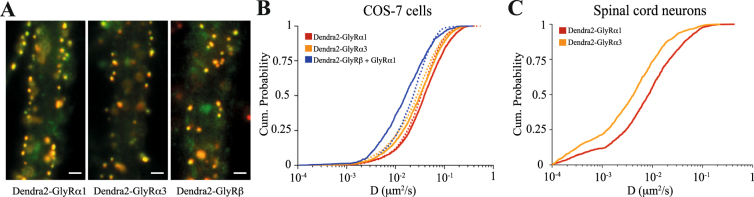
GlyRα subunit dependent protein dynamics at synapses. (**A**) Conventional fluorescence microscopy of spinal cord neurons at DIV14 expressing Dendra2-GlyRα1, α3 or GlyRβ (green) and immunolabelled for the synaptic scaffold protein gephyrin (red). Scale: 1 μm. (**B**) Cumulative distribution of diffusion coefficients of QD-tagged Dendra2-GlyRs in COS-7 cells. SPT was carried out in transfected cells expressing GlyRs alone (dotted lines) or together with mRFP-gephyrin (solid lines). The presence of gephyrin decreased the speed of diffusion of heteromeric GlyRs formed by Dendra2-GlyRβ and untagged α1 subunits (blue) significantly (MW p < 0.0001), whereas homomeric Dendra2-GlyRα1 (red) and α3 receptors (orange) were not affected (p = 0.28 and 0.09, respectively). Statistical analysis was done using the median D values of the movies (n > 30), representing over 1200 trajectories per condition from 3 independent experiments. (**C**) At spinal cord synapses, heteromeric GlyRs composed of endogenous GlyRβ subunits and Dendra2-GlyRα1 (red) display a greater mobility than heteromeric receptors containing Dendra2-GlyRα3 (orange; n > 1350 trajectories, 35–48 cells, 3 experiments).

Earlier studies have shown that the accumulation of glycine receptors at inhibitory synapses depends on direct interactions between GlyRβ subunits and the gephyrin scaffold. To determine whether the α subunits of the receptor also play a part in the synaptic clustering we compared the tendency of α1 and α3 containing GlyRs to accumulate at synapses. Spinal cord neuron cultures were infected at DIV5 with lentivirus driving the expression of N-terminally-tagged Dendra2-GlyRα1, α3 or β, fixed at DIV14 and immunolabelled for gephyrin. All subunits co-localised with synaptic gephyrin puncta, indicating that the receptors bind to the synaptic scaffold (Fig. 1A). As a measure of this interaction we calculated the intensity correlation quotient (ICQ) between receptor and gephyrin fluorescence^17^. Dendra2-GlyRα1 and α3 had a similar propensity to cluster at gephyrin puncta as Dendra2-GlyRβ (ICQ α1: 0.213 ± 0.020; α3: 0.250 ± 0.013; β: 0.208 ± 0.014; KW p = 0.08). In hippocampal neurons that do not express endogenous β subunits^18^, Dendra2-GlyRα1 and α3 failed to accumulate at gephyrin clusters (Fig. S1), resulting in low ICQ values (α1: 0.114 ± 0.015; α3: 0.102 ± 0.017).

To substantiate these findings, quantum dot (QD) based single particle tracking (SPT) of GlyRα1 and α3 receptors was performed in the presence or absence of gephyrin in COS-7 cells. This reduced model is well suited to reveal interactions between receptors and scaffold proteins in living cells that are seen as changes in GlyR membrane diffusion^5^. As expected, mRFP-gephyrin slowed down the diffusion of heteromeric receptors composed of Dendra2-GlyRβ and untagged GlyRα1 (median D_no geph_: 0.023 µm^2^/s, D_geph_: 0.016 µm^2^/s, n > 30 cells, MW p < 0.0001), but had no influence on homomeric Dendra2-GlyRα1 or α3 receptors (p = 0.28 and 0.09, respectively; Fig. 1B). To characterise the diffusion properties of α1 and α3 containing heteromeric GlyR complexes at synapses, we carried out QD-SPT of recombinant GlyRs in spinal cord neurons (Fig. 1C). Surprisingly, synaptic Dendra2-GlyRα1 containing receptors had a much greater mobility than Dendra2-GlyRα3 (median D, α1: 0.0084 µm^2^/s, α3: 0.0042 µm^2^/s, p < 0.0001), despite the fact that the interaction of both types of receptor with the synaptic scaffold is mediated by the β subunit. This lends support to our hypothesis that the α subunits can indeed shape the dynamic behaviour of GlyRs.

### Stoichiometry of extrasynaptic GlyR and gephyrin complexes

Recent cryo-EM and crystallographic data have shown that GlyRα1 and α3 assemble into homopentameric complexes^19, 20^. There remains controversy regarding the stoichiometry of heteromeric GlyRs, where both α_3_:β_2_ and α_2_:β_3_ models have been proposed (discussed in^21^). Given the implication of the β subunit in gephyrin binding and synaptic clustering, we probed for possible differences of the assembly of α1 and α3 containing heteromeric GlyRs that could explain their different diffusion properties. To this aim we applied PALM based single molecule counting as first described by Durisic and colleagues^22^. In this approach the number of molecules within a complex is determined by counting spatially and temporally separated bursts of detections arising from single fluorophores. A difficulty lies in the fact that only a fraction of the fluorophores is functional^21^. We therefore initially determined the probability of detection p_det_ of Dendra2-tagged GlyRs with a known homopentameric subunit stoichiometry.

**Figure 2:**
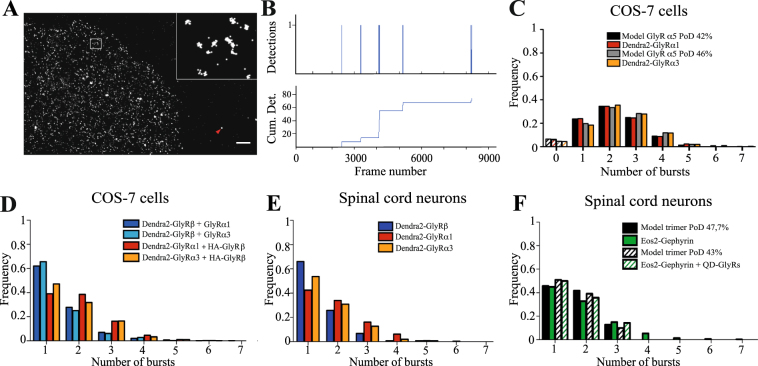
Stoichiometry of extrasynaptic GlyR and gephyrin complexes. (**A**) Pointillist image of Dendra2-GlyRα1 detections in a COS-7 cell after complete photoconversion of all fluorophores. Clusters of detections from individual fluorophores can be spatially resolved (inset). Fluorescence beads were used to correct x/y drifts during acquisition (red arrowhead). Scale: 2 μm. (**B**) Example of a time-trace of detections within a 120 nm cluster (cumulative trace below). Bursts of detections separated by more than 250 frames (12.5 seconds) were considered as discrete photoconversion events. (**C**) Histogram of burst frequencies of homomeric Dendra2-GlyRα1 (red bars) and Dendra2-GlyRα3 complexes (orange). Fitting of the data with a binomial distribution for a pentameric α_5_ GlyR model gave the probability of detection (PoD) p_det_ = 0.42 (black bars) and 0.46 (grey), respectively. The number of missed events (zero bursts) was derived from the binomial fitting (dashed bars; Table S1). (**D**) Histogram of burst frequencies from α and β subunits co-expressed in COS-7 cells. The distributions of Dendra2-GlyRα1 (red) or α3 (orange) co-expressed with untagged β subunits are skewed towards higher values compared to Dendra2-GlyRβ with either of the two untagged α subunits (dark and light blue). (**E**) The burst frequencies of Dendra2-tagged α1 (red), α3 (orange) and β subunits (blue) in extrasynaptic regions of infected spinal cord neurons point to an α_3_:β_2_ stoichiometry of heteromeric GlyRs (raw counts and fitting results are given in Table S1). (**F**) Bursts of mEos2-gephyrin detections in infected spinal cord neurons (green) and the closest fit with a model of three molecules and p_det_ = 0.48 (black). Similar results were obtained for mEos2-gephyrin clusters associated with QD-tagged endogenous GlyRs in the extrasynaptic plasma membrane (dashed bars; Table S2).

Dendra2-GlyRα1 or α3 were first expressed in COS-7 cells that do not contain endogenous GlyRs or gephyrin. PALM imaging was done in fixed cells with low levels of recombinant receptors, using fiducial markers to correct for x/y drift during acquisition (Fig. 2A). Clusters of detections within a diameter of 120 nm were selected with a homemade plugin in Matlab (CountMol), applying a density threshold to remove the background of single detections. Bursts of detections were considered to stem from the same fluorophore when they occurred in a time window of less than 250 frames ( < 12.5 s; Fig. 2B), within which over 95% of fluorophores were bleached. The histogram of the burst counts was fitted with a binomial distribution assuming pentameric complexes, where the total number of clusters N and the probability of detection p_det_ were free variables (Table S1). According to this analysis, α1 and α3 did not differ in their assembly as homopentameric receptors (see also Fig. S1). The p_det_ of Dendra2 was 42% for GlyRα1 and 46% for GlyRα3 (Fig. 2C), in line with earlier estimates (p_det_ = 0.48^22^).

We then co-expressed Dendra2-tagged GlyRα1, α3 or β together with non-fluorescent subunits in COS-7 cells to determine the stoichiometry of heteromeric GlyRs. The distribution of bursts of Dendra2-GlyRα1 and α3 was skewed towards higher numbers compared to Dendra2-GlyRβ containing complexes, indicating that there are more α than β subunits per pentamer (Fig. 2D). When comparing the measured frequencies with the theoretical binomial distribution for an average p_det_ = 0.44 for Dendra2, the lowest residuals were obtained for a model containing two β subunits and three α3 subunits (Table S1). The distribution of α1 counts could be explained by three or by four subunits per complex, possibly due to some overcounting and/or the presence of a small population of homopentameric GlyRα1 (see discussion).

Experiments were also done on infected spinal cord neurons expressing Dendra2-tagged GlyRα1, α3 or β (Fig. 2E). Numbers of bursts were counted in clusters of detections within sparse areas of the extrasynaptic plasma membrane. We found virtually the same distribution of burst frequencies as in COS-7 cells, confirming that the recombinant receptors co-assembled with their endogenous counterparts with an α_3_:β_2_ stoichiometry, irrespective of the type of α subunit (Table S1). Based on our cluster selection parameters, we derived apparent densities of 3.5 ± 0.8 α1 and 3.1 ± 0.5 α3-containing complexes per µm^2^ in the extrasynaptic membrane. Since the p_det_ accounts for the population of non-fluorescent subunits, the similar burst distributions also suggest that endogenous α subunits are replaced by the overexpression of recombinant α1 and α3. This is supported by immunolabelling in Dendra2-GlyRα1 and α3 expressing neurons in which around 80% of the endogenous GlyRs are replaced (Fig. S2). Furthermore, lentiviral expression of α subunits did not increase the total GlyR levels at synapses compared to non-infected neurons (Fig. S3).

We similarly counted the stoichiometry of mEos2-gephyrin oligomers in COS-7 cells (cluster diameter 150 nm, burst length < 60 s). The basic unit of gephyrin is believed to be that of a trimer according to crystallographic data^23, 24^, although some evidence points to the existence of hexameric gephyrin complexes^25, 26^. Fitting the burst frequencies with the binomial distribution of a trimer resulted in p_det_ = 0.52 that is in agreement with earlier estimates (p_det_ = 0.61^22^). We obtained some higher values (k > 3) that could result from overcounting or from the presence of higher order oligomers. As a consequence, the data could also be fitted with a hexameric model, however, this was rejected based on the exceedingly low p_det_ = 0.28 associated with this solution (Table S2).

Single molecule counting of mEos2-gephyrin in neurons gave a similar overall distribution, confirming that non-clustered gephyrin mostly forms trimers (Fig. 2F). The somewhat lower p_det_ = 0.48 compared to COS-7 cells is likely due to the presence of endogenous gephyrin in spinal cord neurons that dilutes the number of detected mEos2-gephyrin molecules. For a selective analysis of membrane-associated mEos2-gephyrin, QDs were attached via specific antibodies to the extracellular epitope of endogenous GlyRα1 (see methods). Fitting of the mEos2-gephyrin burst frequencies at QD sites with a binomial distribution for a model of ‘three’ returned a probability of p_det_ = 0.43 (Fig. 2F, Table S2). QDs without associated mEos2 detections represented 44% of cases and were excluded from the fit. These data confirm that more than half of the extrasynaptic GlyRs are bound to gephyrin^8^, and allow the conclusion that receptor complexes associate with gephyrin trimers in a 1:1 stoichiometry outside of synapses.

### Absolute numbers and densities of GlyRα1 and α3 at spinal cord synapses

Beyond quantifying subunit stoichiometry, single fluorophore counting can be used to estimate the total molecule numbers at synapses^21^. Knowing p_det_ is essential for this purpose, since this parameter not only describes the photo-physical properties of the fluorophores, but also discloses the invisible, endogenous molecule fraction^22^. We calculated the numbers of receptor subunits at inhibitory synapses by converting the number of detections at synaptic clusters into the number of bursts, and further correcting the data for the non-detected fraction and receptor stoichiometry.

**Figure 3:**
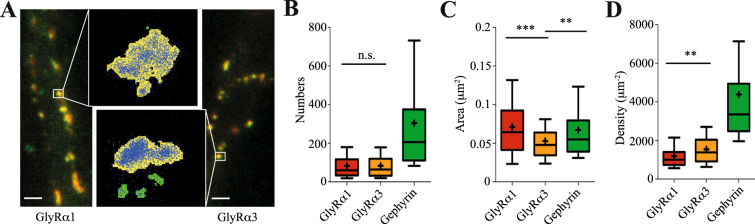
Absolute numbers and densities of GlyRα1 and α3 at spinal cord synapses. (**A**) Conventional fluorescence microscopy of spinal cord neurons expressing Dendra2-GlyRα1 or α3 (green) and stained for endogenous gephyrin (red). Scale: 2 μm. Insets: Pointillist images of GlyRα1 and α3 detections (blue) at synaptic clusters (density-thresholded yellow areas). (**B**) Absolute numbers of Dendra2-tagged α1 (red) and α3 (orange) containing GlyR complexes at synapses (83 ± 5 mean ± SEM for both subunits; MW p = 0.86, n ≥ 170 clusters per conditions from > 15 cells and 7 independent experiments). mEos2-gephyrin molecules were independently quantified in different neurons (green; 304 ± 29 molecules, n > 120 clusters, 12 cells, 3 experiments). (**C**) Receptor and scaffold clusters sizes range from 0.01 to 0.15 μm^2^. GlyRα3 clusters were on average smaller than both GlyRα1 (MW p < 0.0001) and mEos2-gephyrin clusters (p < 0.01). (**D**) Densities of receptors and scaffold proteins were derived from the absolute molecule numbers and cluster size. GlyRα3 containing receptors were clustered more densely than GlyRα1 (MW p < 0.0001).

We analysed dense clusters of Dendra2-GlyRα1 or α3 detections in pointillist images of spinal cord neurons that corresponded to focussed gephyrin positive puncta in conventional fluorescence images (Fig. 3A). The total number of detections per cluster was divided by the mean detections per Dendra2 burst (13–19 depending on the recording) and by p_det_. This yielded a total of 250 copies of GlyRα1 subunits per synapse, which equals 83 ± 5 heteromeric GlyR complexes (Fig. 3B). We obtained the same value for GlyRα3, indicating that both subunits occupy the same number of binding sites under control conditions. The gephyrin levels were not different at α1 and α3 positive synapses in these cells, judging from the background corrected total fluorescence intensity (Fig. 3A; 504 ± 59 and 588 ± 42 arbitrary units × 10^2^, respectively; n = 170–177 clusters, MW p = 0.311). The independent quantification of mEos2-gephyrin detections in spinal cord neurons gave a mean of 304 ± 29 molecules per synaptic cluster, which suggests an occupancy of up to half the receptor binding sites assuming an α_3_:β_2_ receptor stoichiometry.

Making use of the high spatial resolution of PALM we compared the areas occupied by GlyRα1, α3 and gephyrin (Fig. 3C). Remarkably, GlyRα3 detections were concentrated within a significantly smaller area (0.052 ± 0.002 µm^2^) than GlyRα1 (0.071 ± 0.004 µm^2^; MW p < 0.0001). Consequently, the derived two-dimensional densities of GlyRα3 containing receptors (1546 ± 61 complexes µm^−2^) were higher than those of GlyRα1 (1186 ± 51 µm^−2^, Fig. 3D). This indicates that the clustering of GlyRs is indeed shaped by the α subunits, as are their diffusion properties at synapses (Fig. 1C). In independent neurons, the size of mEos2-gephyrin clusters (0.067 ± 0.004 µm^2^) closely matched that of GlyRα1 clusters (p = 0.25), resulting in a two-dimensional density of 4381 ± 531 gephyrin molecules per µm^2^.

### Regulation of endogenous GlyR levels at synapses by interleukin 1β

**Figure 4:**
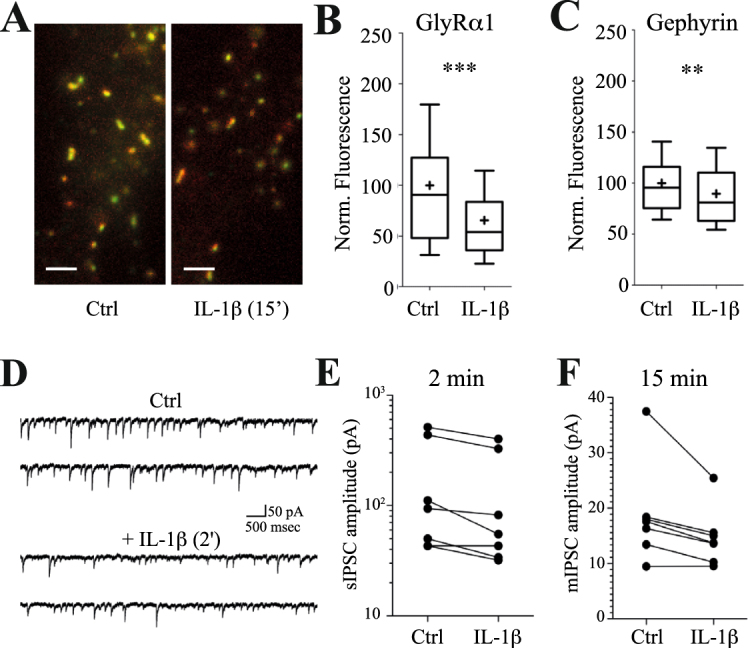
Regulation of endogenous GlyR levels at synapses by interleukin 1β. (**A**) Fluorescence images of spinal cord neurons (DIV14) stained for endogenous GlyRα1 (green) and gephyrin (red) in control condition and after 15 minutes of IL-1β application. Scale: 2 μm. (**B**) GlyRα1-associated integrated fluorescence intensity at gephyrin clusters after 15 min application of 10 ng/ml IL-1β was reduced by 34% compared to control (n ≥ 95 cells per condition from 3 experiments; MW p < 0.0001). (**C**) Gephyrin fluorescence intensity at synapses in control and IL-1β treated neurons (MW p < 0.01). (**D**) Representative whole-cell recording of spontaneous glycinergic currents from a DIV21 spinal cord neuron under control conditions (top) and after 2 min application of IL-1β (bottom). (**E**) A reduction of the mean sIPSC amplitude by IL-1β was seen in 6 out of 7 neurons (Wilcoxon test p < 0.05). (**F**) In independent recordings the mean mIPSC amplitudes were decreased in 6 out of 7 spinal cord neurons after 15 min perfusion with IL-1β (Wilcoxon test p < 0.05).

The impact of the α subunits on the clustering and diffusion properties of GlyRs raises the possibility that regulatory mechanisms acting on receptor function may trigger subunit-specific receptor redistribution. We therefore challenged the system with the cytokine IL-1β that is known to differentially affect glycinergic currents in distinct populations of neurons in the spinal cord^14, 15^. Bath application with IL-1β led to a reduction of the endogenous levels of GlyRs at synapses in cultured spinal cord neurons (Fig. 4A). The background corrected fluorescence intensity of immunolabelled GlyRα1 was lessened by 34% after 15 minutes of treatment with 10 ng/ml IL-1β (Fig. 4B). Gephyrin labelling was decreased by only 10% under these conditions (Fig. 4C), indicating that the lower GlyR levels were primarily caused by a change in receptor behaviour rather than gephyrin clustering as such.

Electrophysiological measurements showed that IL-1β rapidly reduced the amplitudes of glycinergic spontaneous inhibitory postsynaptic currents (sIPSC; Fig. 4D). This effect was observed in 6 out of 7 neurons, despite a large variability in sIPSC amplitudes in cultured spinal cord neurons that ranged from 30 to 500 pA (Fig. 4E). On average, sIPSC amplitudes decreased by about 28% within 2 minutes of IL-1β application. In an independent set of experiments, we measured the GlyR responses during 15 minute application of IL-1β in the presence of TTX to isolate miniature currents. In 6 out of 7 neurons mIPSC amplitudes were reduced by an average of 21% (Fig. 4F), paralleling the reduction of GlyR levels at synapses. Together, these data show that mechanisms acting on the function of the GlyR may be accompanied by changes in receptor clustering at synapses.

### GlyRα1 specific regulation of receptor numbers and clustering by IL-1β

**Figure 5:**
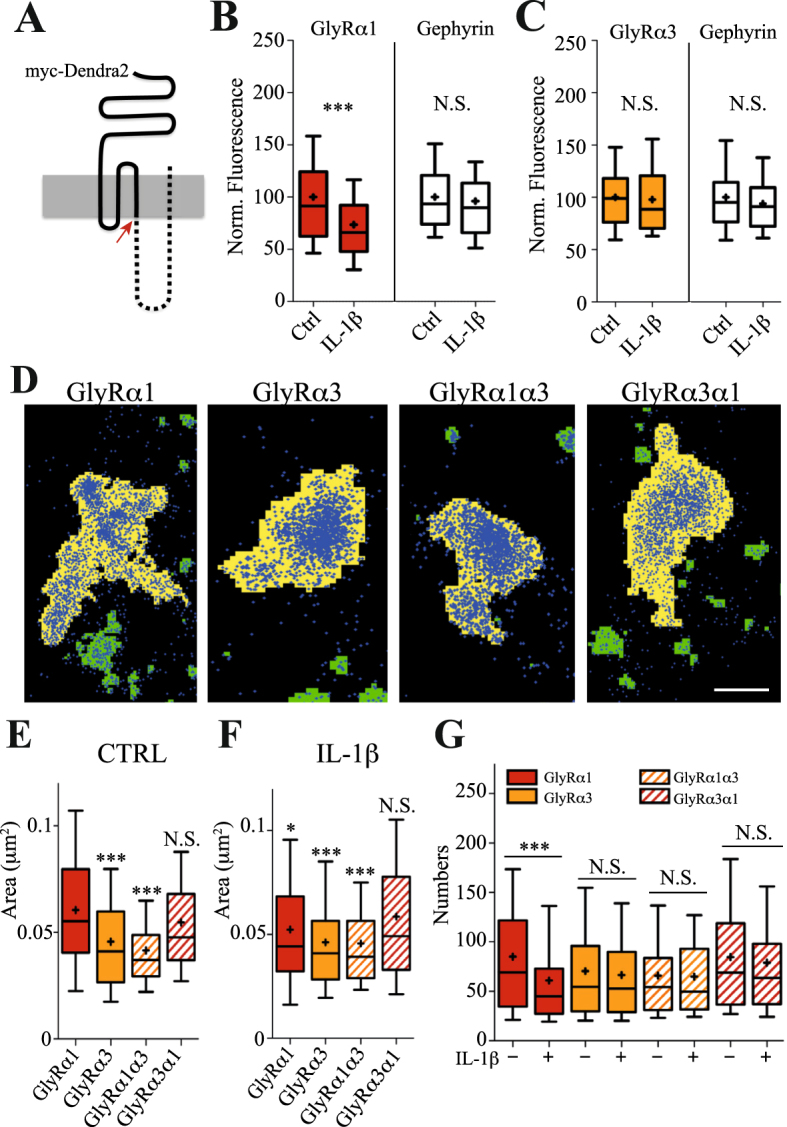
GlyRα1 specific regulation of receptor numbers and clustering by IL-1β. (**A**) Schematic representation of Dendra2-tagged GlyR variants. The cytoplasmic loop and TM4 domain (dotted line) is the most variable region between the α1 and α3 subunits. The red arrow indicates the site at which α1 and α3 sequences were exchanged to create chimeric subunits. (**B**) Application of IL-1β for 15 minutes significantly reduced the integrated fluorescence of Dendra2-GlyRα1 compared to the control (MW p < 0.0001), whereas endogenous gephyrin levels were not affected (MW p = 0.12). (**C**) Integrated fluorescence intensities of Dendra2-GlyRα3 and gephyrin at synapses were not altered after 15 min application of IL-1β (MW p = 0.44 and 0.37, respectively). (**D**) Pointillist images of Dendra2-tagged α1, α3 and the chimeric subunits α1α3 and α3α1 (blue) at synaptic clusters (yellow regions). Scale: 200 nm. (**E**) The sizes of α3 and α1α3 clusters were significantly smaller than those of α1 and α3α1 containing GlyRs (n > 100 clusters, 3 experiments, MW p < 0.0001 versus GlyRα1). (**F**) IL-1β application for 15 min slightly reduced GlyRα1 cluster size (MW p = 0.014),but did not affect the areas of α3, α1α3 and α3α1 constructs when compared to their non-treated control. The statistical analysis reported in (**F**) refers to pairwise comparison of all conditions with the GlyRα1 non-treated control. (**G**) PALM-based quantification of absolute receptor numbers at synapses under control conditions and after 15 min IL-1β treatment. The average number of α1 containing GlyR complexes at synaptic clusters was reduced from 85 ± 5 to 61 ± 4 by IL-1β (mean ± SEM, n = 130 clusters, 3 experiments; MW p < 0.001), whereas α3, α1α3 and α3α1 numbers were not affected.

Given that the effect of IL-1β on endogenous α3 containing GlyRs could not be assessed due to the lack of suitable antibodies, we undertook experiments with recombinant Dendra2-tagged GlyR variants (Fig. 5A). First, we measured the effect of IL-1β on infected spinal cord neurons expressing either Dendra2-GlyRα1 or α3. IL-1β diminished the background-corrected synaptic Dendra2-GlyRα1 fluorescence by 26% compared to control (Fig. 5B), similar to endogenous GlyRα1 (Fig. 4B). Unexpectedly, Dendra2-GlyRα3 levels remained unaltered during IL-1β application (Fig. 5C), pointing to a subunit-specific regulation.

Using PALM-based molecule counting, we then compared the behaviour of GlyRα1 and α3 with that of chimeric constructs in which the cytoplasmic loop and the fourth transmembrane domain (TM4) of α1 and α3 were exchanged with one another (Fig. 5A,D). We measured the synaptic areas occupied by GlyRα1 and α3 as well as the chimeric receptors in the super-resolution PALM images (Fig. 5D,E). This revealed that the cytoplasmic loop and TM4 region had a striking impact on the receptor distribution at synapses. The clusters formed by the chimera comprising the C-terminus of GlyRα1 had a similar size than those of the wild-type α1 subunit (α1_Ctrl_: 0.060 ± 0.003 µm^2^, α3α1_Ctrl_: 0.055 ± 0.003 µm^2^; MW p = 0.14). In the same way, the C-terminus of α3 induced the formation of smaller and denser clusters of GlyRα3 and the α1α3 chimera (α3_Ctrl_: 0.046 ± 0.002 µm^2^, α1α3_Ctrl_: 0.041 ± 0.002 µm^2^; MW p = 0.40; versus α1: p < 0.0001). This points to a specific role of the cytoplasmic and TM4 sequences of the α subunits in the synaptic distribution of GlyRs.

Application of IL-1β had no obvious effect on the described pattern of cluster sizes (Fig. 5F), despite a small reduction in the size of GlyRα1 clusters (α1_Ctrl_: 0.060 ± 0.003 µm^2^, α1_IL-1β_: 0.052 ± 0.003 µm^2^; MW p = 0.014). This, however, is likely the result of the pronounced α1-specific dispersal of GlyRs from synapses. In absolute numbers, IL-1β application led to the removal of about 20 α1 complexes from an average synapse (Fig. 5G). IL-1β had no effect on α3 or on chimeric α1α3 and α3α1 receptors at synapses, indicating that the regulation of GlyR numbers requires both the extracellular domain as well as the cytoplasmic loop of α1 (Fig. 5G).

## Discussion

In this study, we tested the hypothesis that the GlyRα subunits influence the receptor clustering at synapses, which was thought to depend exclusively on the interaction of the β subunit with the gephyrin scaffold. To this aim, we applied a novel quantitative imaging approach comprising single molecule localisation, counting and tracking, to characterise the behaviour of receptor complexes starting from their basic subunit composition, to the impact of a regulatory mechanism on absolute receptor numbers at synapses. We found clear evidence that α1 and α3 subunits modulate the dynamic equilibrium of GlyRs in spinal cord neurons even though they share the same heteropentameric stoichiometry and thus the same gephyrin binding capacity.

GlyR subunits α1 and α3 are known to assemble as homopentameric complexes^19, 20^. Despite substantial efforts, the stoichiometry of heteromeric GlyRs remains a matter of dispute. Whereas most biochemical and electrophysiological data point to an α_3_:β_2_ stoichiometry^27–29^, others support an α_2_:β_3_ model^30, 31^, with little to choose between them. A recent study applying fluorophore counting to dissect the GlyR stoichiometry could provide some closure to this debate^32^. Judging from bleaching steps of single YFP fluorophores attached to the cytoplasmic loop of GlyR subunits expressed in *Xenopus laevis* oocytes the authors argued for heteromeric α_3_:β_2_ complexes. Using PALM-based molecule counting we now provide evidence that GlyRα1 as well as α3 subunits assemble with β subunits in a 3:2 ratio in spinal cord neurons. Our approach is comparable to the bleaching step method in that both rely on the detection of single fluorophores and the estimation of the invisible fraction through binomial fitting^22^. The use of inclined laser illumination rather than TIRF, and the choice of mature cultured neurons make these experiments challenging. In particular, over- and under-counting of fluorophores constitute the main source of error^21^, while the fitting of very few data points may produce inconclusive results (discussed in the supplement of ^33^). Despite these technical difficulties our data clearly favour the α_3_:β_2_ model of GlyR assembly. The most likely internal arrangement is one of alternating subunits (α-β-α-β-α), since this requires α-β, β-α and α-α interactions, but excludes the formation of a β-β interface. It is thus compatible with the observation that α subunits can form functional homomeric receptors whereas β subunits do not^28, 34^. Accordingly, the existence of complexes with an α_4_:β_1_ stoichiometry (as proposed in^27^) cannot be formally excluded, but is unlikely given our results.

Previous models describing diffusion-trapping processes of membrane receptors at synapses account for both receptor-scaffold as well as scaffold-scaffold interactions (e.g.^35^). Such a situation exists at inhibitory synapses, where diffusing GlyRs are immobilised through the interaction with the scaffold protein gephyrin. Our data largely confirm the dominant role of the β subunit in the regulation of GlyR-gephyrin binding. We did not observe a direct interaction between α subunits and gephyrin, implying that GlyR clustering must be mediated by the β subunit. Furthermore, α1 and α3 containing complexes displayed the same tendency to accumulate at synapses, as expected for heteromeric receptors that contain a common β subunit controlling gephyrin binding. Taking a PALM-based molecule counting approach our study offers a quantitative view of receptor accumulation at synapses. This type of information can help to calculate absolute molecule fluxes and binding constants, and ultimately estimate thermodynamic parameters in living cells^6^. We found that about 80 GlyR complexes are clustered at spinal cord synapses. This compares to an average of 300 gephyrin molecules, meaning that GlyRs occupy up to half of the synaptic binding sites at steady state, considering their α_3_:β_2_ stoichiometry. The remaining sites may be available for diffusing receptors or occupied by other binding partners such as GABA_A_Rs. An interesting concept that may be inferred from our data is that extrasynaptic GlyRs behave similarly to the synaptic receptors as regards their composition and basic gephyrin binding properties. The identity of synaptic and extrasynaptic populations of ionotropic ion channels is an assumption that is often made when receptor numbers are estimated by electrophysiological means^36^. Our data affirm that extrasynaptic GlyRs are α_3_:β_2_ heteromers, a majority of which are bound to a trimer of gephyrin.

Our quantification of synaptic GlyRs exceeds the numbers of available receptors deduced from mIPSC measurements in rat motoneurons and in zebrafish hindbrain (~40 GlyRs^36, 37^). This could simply be due to the use of widely different experimental systems. Nonetheless, it raises the question whether the overexpression of recombinant GlyRs affects the receptor numbers at inhibitory synapses in cultured spinal cord neurons. There are several arguments that this is not the case. Firstly, the p_det_ accounts for the non-fluorescent fraction of molecules and therefore includes the endogenous subunit population. Secondly, about 80% of endogenous GlyRα1 is replaced by recombinant α subunits (Fig. S2). Also, total GlyR levels are not increased in neurons expressing recombinant α1 subunits compared to non-infected controls (Fig. S3). Earlier experiments have shown that overexpression of gephyrin similarly replaces the endogenous protein without substantially affecting copy numbers at synapses (~200 molecules not considering p_det_^38^). In other words, the composition of inhibitory synapses appears to be relatively resistant to changes in the expression of individual constituents. Saying that, we did observe that upon lentiviral expression of GlyRα subunits the immunoreactivity of endogenous gephyrin and GABA_A_Rβ3 was reduced compared to non-infected neurons (Figs S2 and S3). While this suggests that the GlyR occupancy of synaptic binding sites is indeed altered in infected cells, it has no bearing on our quantification of absolute GlyR numbers, nor on the comparison between different α subunit variants and their regulation.

Receptor-gephyrin binding is regulated by post-translational modifications targeting either the gephyrin scaffold^3, 39^ or the binding domain of the receptor itself^4, 5^. Our discovery of an α subunit dependent modulation of GlyR clustering adds a further twist to the dynamic equilibrium at inhibitory synapses. The new data demonstrate that GlyRβ-gephyrin binding is influenced by the α subunits of heteromeric receptors, too. In line with earlier recordings in spinal cord slices^14^, application of IL-1β rapidly reduced sIPSC amplitudes as well as GlyR numbers at synapses. The timing of the observed changes suggests that GlyR redistribution follows the decrease in receptor function in response to IL-1β. Even though the mechanism by which IL-1β acts on GlyRs is not known, these findings imply that receptor function and gephyrin binding are not independent properties. Instead, we think that conformational changes triggered at extracellular^19, 20, 40^ or intracellular protein domains^41^ have downstream consequences on channel opening as well as receptor clustering. This is reminiscent of data showing that the dynamic properties of GABA_A_Rs and AMPARs can be modulated by ligands that induce conformational (and functional) changes^16, 42^. An alternative explanation could be that other proteins (e.g. sec 8) interact with GlyRs in an α subunit-specific manner, similar to radixin-dependent GABA_A_Rα5 clustering^43, 44^. However, we believe that this is less likely, since the effect of IL-1β was not transferred from α1 to either of the two chimeric subunits.

We thus propose that the trapping and subsequent accumulation of GlyRs at synapses is controlled by an inter-molecular crosstalk between α and β subunits. Remarkably, IL-1β affects α1 but not α3 containing GlyRs, showing that they transmit different signals to the gephyrin binding site. This difference is evident also in the subunit specific clustering of GlyRs at synapses, where α1 containing receptors are more mobile and packed less densely. Our analysis of the chimera further demonstrates that GlyR clustering depends on the C-terminal domains of the α subunit, whereas the effect of IL-1β implicates also the extracellular regions of α1. These findings predict that GlyR subtypes are differentially regulated by IL-1β, which could contribute to cell-type specific processes in the context of inflammatory pain. Such a cell-type specificity may underlie the opposite effects of IL-1β on GlyR currents in different populations of spinal cord interneurons, where either inhibition or the potentiation of glycinergic currents have been reported^14, 15^. Our results therefore highlight the importance of studying defined neuron populations expressing known receptor subtypes.

## Methods

### Expression constructs

The coding sequences of rat GlyRα1 (UniProt P07727–1, isoform a), human GlyRα3L (UniProt O75311–1, long isoform), and human GlyRβ (UniProt P48167–1) were inserted into an FUGW replicon containing the signal peptide of GlyRα1 fused with a myc tag and Dendra2, to generate FU-SP-myc-Dendra2-GlyRα1, α3L, β, as well as the two chimeras α1α3L and α3Lα1. The plasmid FU-mEos2-gephyrin was derived from an earlier construct^38^. The replicon plasmids were used for transfection of COS-7 cells as well as lentivirus production.

### Cell culture and treatment

COS-7 cells were cultured and transfected using FuGENE 6 as described previously^5^. Primary cultures of spinal cord neurons were prepared from embryonic Sprague Dawley rats at day 14 (E14) and mRFP-gephyrin knock-in (KI) mice at E13 as described before^38^, in accordance with guidelines of the French Ministry of Agriculture and the Direction Départamentale des services véterinaires de Paris (Ecole Normale Supérieure, animalerie des rongeurs, license B 75–05–20). Neurons were infected with lentivirus at 1–5 days *in vitro* (DIV) if required, and used for experiments at DIV 14–21. IL-1β (Sigma) was applied in culture medium for 15 minutes at a final concentration of 10 ng/ml prior to fixation and immunolabelling, or added to the external solution for electrophysiological recordings.

### Immunolabelling

Fixed cells were labelled with primary antibodies (rabbit anti-GlyRα1, custom made; mouse anti-gephyrin mAb7a, Synaptic Systems 147011) and secondary antibodies (Cy3-conjugated goat anti rabbit IgG and Alexa Fluor 647 goat anti mouse). For PALM counting of receptor-associated mEos2-gephyrin (Fig. 2F), mouse spinal cord neurons were fixed and endogenous GlyRs were labelled with QDs emitting at 605 nm (primary antibody: mouse anti-GlyRα1, Synaptic Systems mAb2b; secondary antibody: anti-mouse Fab’-QD_605_).

### Fluorescence image acquisition and analysis

Images were acquired with NIS software on an inverted Nikon Eclipse Ti microscope with a 100x oil-immersion objective (NA 1.49), an additional 1.5x lens, a mercury lamp and an Andor iXon EMCCD camera (16 bit, image pixel size 107 nm), using specific emission filters (525 nm with 30 nm bandwidth for non-converted Dendra2 and mEos2; 607/36 for mRFP and Cy3; 684/24 for Alexa Fluor 647). Cluster analysis was done in selected regions of interest by creating binary masks of gephyrin clusters using image segmentation^45^ in Metamorph (Molecular Devices). Background corrected, integrated GlyR and gephyrin fluorescence intensities were measured in the clusters of the mask. Intensity correlation analysis (ICA) was used to quantify the correlation between Dendra2-GlyR and gephyrin labelling at gephyrin clusters^17^. High intensity correlation quotient values (0 < ICQ ≤ 0.5) indicate positive correlation between the fluorescence in the two channels.

### Single-particle tracking

The diffusion of Dendra2-tagged GlyRs in COS-7 cells or spinal cord neurons was measured by single-particle tracking (SPT) using quantum dots (QDs) as described^5^. Cells were labelled with Dendra2 antibody (Antibodies-online ABIN361314), biotinylated anti-rabbit F(ab’)_2_ and streptavidin coated QDs emitting at 655 nm, and imaged for up to 30 min. QD trajectories (500 frames at 13 Hz) were analysed using homemade software in Matlab (Mathworks). Diffusion coefficients D were calculated by fitting the first five points of the mean square displacement (MSD) plot against time.

### Photoactivated localisation microscopy (PALM)

Fixed cells were imaged in PBS in an open chamber on the Nikon microscope described above. PALM movies of up to 20000 frames were acquired at 20 Hz with inclined 561 nm illumination with 30% (Dendra2) or 40% (mEos2) of 500 mW laser output and a specific emission filter (607/36). Photoconversion was done by pulsed 405 nm illumination (0.5 ms pulses) at increasing power to ensure complete conversion of all fluorophores by the end of the recording. The z position was maintained during acquisition using a Nikon perfect focus system. PALM movies were analysed by fitting the point-spread function of each fluorophore with a 2D Gaussian distribution^46^. The x/y drifts were corrected during analysis with fiducial markers (100 nm fluorescent TetraSpeck beads, Thermo Fisher).

To count gephyrin molecules associated with extrasynaptic GlyRs (Fig. 2F), QDs were first localised by continuous illumination at 561 nm (20% laser power) for 1000 frames and subsequently bleached by applying 561 nm laser for several minutes (100% laser power). mEos2-gephyrin was then imaged as described above, and the QD and mEos2 recordings fused into a single stack for analysis.

### PALM-based molecule counting

To automatically select and analyse the intensity-time traces of photoconverted fluorophores in the pointillist image, we developed a software written in MATLAB (CountMol). First, drift corrected pointillist images were rendered by representing each detection with a Gaussian distribution (σ = 10 nm). A density-based threshold was applied to remove single detection noise. Clusters of detections of up to 100 nm in diameter with at least 9 detections were automatically selected, a circular mask of 120 nm (Dendra2) or 150 nm (mEos2) in diameter was superimposed to these pre-selected clusters, and the detections within the mask were automatically counted. In pulsed photoconversion experiments we determined a maximal bleaching time τ_bleach_ of 12.5 s (250 frames) for Dendra2 fluorophores and of 60 s (1200 frames) for mEos2. Accordingly, τ_bleach_ was used as a cutoff to discriminate between bursts of detections belonging to the same or separate fluorophores.

To determine the number of detections at synapses, a stringent density-based threshold was applied to remove detections not belonging to synaptic clusters. In addition, only clusters of detections that had a corresponding receptor/gephyrin punctum in the conventional fluorescence image were taken into account. The absolute number of molecules *N* in the cluster was calculated with the equation:

$$N=x/np$$

where *x* is the total number of detections in the cluster, *n* is the average number of detections per fluorophore, and *p* is the probability of detection of the fluorophore (Dendra2: *n* = 13–19; *p* = 0.44; mEos2: *n* = 19; *p* = 0.48). To calculate the absolute number of receptor complexes in the cluster, *N* was divided by the number of subunits in the heteropentamer according to the receptor stoichiometry.

### Electrophysiology

Whole-cell patch-clamp recordings were performed at room temperature in voltage-clamp mode using a Multiclamp 700B controlled by pClamp 10 acquisition software (Molecular Devices). Currents were filtered at 2–3 kHz and sampled at 10 kHz using a Digidata 1440 A (Molecular Devices). Patch pipettes (4–6 MΩ) were filled with internal solution containing 70 mM CsCl, 70 mM caesium methanesulphonate, 1 mM EGTA, 1 mM MgCl_2_, 4 mM Mg-ATP and 10 mM HEPES, adjusted to pH 7.4 with CsOH. Spontaneous glycinergic currents were recorded at a holding potential *V*_*H*_ = −60 mV in external solution containing 137 mM NaCl, 5 mM KCl, 2 mM CaCl_2_, 1 mM MgCl_2_, 20 mM glucose and 10 mM HEPES, pH 7.4. AMPARs, NMDARs and GABA_A_Rs were blocked with 5 µM NBQX, 50 µM D-APV and 5 µM gabazine. In an independent set of experiments (data in Fig. 4F) 0.5 µM tetrodotoxin (TTX) was added to the external solution to isolate miniature currents (mIPSCs) and recordings were done at 32 °C. Spontaneous and miniature glycinergic currents were detected using the Clampfit template procedure. For each cell a sliding template was created by averaging 15–20 events selected by eye, and detection of synaptic currents was done on the basis of closeness of fit to the template. Overlapping events were excluded from analysis.

### Statistical analysis

Immunofluorescence, PALM and QD-SPT data were analysed using a non-parametric Mann-Whitney U test (MW) or Krustal-Wallis non-parametric test with Dunnett’s post test (KW). Electrophysiological data were analysed with a non-parametric Wilcoxon matched-pairs signed rank test. Data are generally expressed as mean ± SEM unless stated otherwise. In bar graphs, the data range is represented as the 10%, 25%, 50% (median), 75% and 90% of the population (shown as boxes and vertical lines). Mean values are indicated by a plus sign.

The PALM counting data (Tables S1 and S2) were fitted with a binomial distribution:

$${{\rm{p}}}_{{\rm{k}}}=[{\rm{n}}!/{\rm{k}}!({\rm{n}}-{\rm{k}})!]{{\rm{p}}}_{{\rm{\det }}}^{{\rm{k}}}{(1-{{\rm{p}}}_{{\rm{\det }}})}^{n-k}$$

where p_k_ is the probability to count k bursts of detection in the time trace, n is the number of subunits of the molecular complex, and p_det_ is the probability to detect the fluorophore. The goodness of the fit was tested with a chi-square test

$${{\rm{\chi }}}^{2}={{\sum }^{{\rm{n}}}}_{{\rm{k}}}{[({{\rm{x}}}_{{\rm{k}}}-{{\rm{X}}}_{{\rm{k}}})/\surd {{\rm{X}}}_{{\rm{k}}}]}^{2}$$

where k is the number of bursts, x_k_ is observed burst frequency and X_k_ is the expected frequency. Fitting assumes a Poisson distribution of the error $\surd {{\rm{X}}}_{{\rm{k}}}$. Values of ${{\rm{\chi }}}^{2}$close to the number of the degrees of freedom F indicate good correspondence between the observed and expected counts $({{\rm{\chi }}}^{2}\approx {\rm{F}}\pm \surd 2{\rm{F}})$.

### Data availability

All data generated or analysed during this study are included in the published article and its supplementary information files. Datasets are available from the corresponding author upon request.

## Electronic supplementary material

Supplemental information
