# Supplementary material for: Alpha subunit-dependent glycine receptor clustering and regulation of synaptic receptor numbers
Source: Sci Rep. 2017 Sep 7;7:10899. doi: 10.1038/s41598-017-11264-3 (PMC5589798; doi:10.1038/s41598-017-11264-3)
Supplement: Supplementary file 1 — Supplemental information [file 41598_2017_11264_MOESM1_ESM.pdf]

Supplemental information

Alpha subunit-dependent GlyR clustering and regulation of synaptic receptor numbers

Patrizio A[1], Renner M[2], Pizzarelli R[1], Triller A[1], Specht CG[1]

[1] École Normale Supérieure, PSL Research University, CNRS, Inserm, Institute of Biology (IBENS), Paris 75005, France

[2] Université Pierre et Marie Curie, Institut du Fer à Moulin (IFM), Paris 75005, France

Supplemental experimental procedures

Expression constructs

The signal peptide of rat GlyRα1 (UniProt P07727-1) was fused to a myc tag (EQKLISEEDL) and Dendra2 (Clontech 632546) and cloned into the FUGW replicon [1] to create FU-SP-myc-Dendra2. The coding sequences of rat GlyRα1 (UniProt P07727-1, isoform a), human GlyRα3L (UniProt O75311-1, long isoform; cDNA provided by V. Eulenburg, Institut für Biochemie, Erlangen), and human GlyRβ (UniProt P48167-1) without their signal peptides were inserted into this backbone to generate FU-SP-myc-Dendra2-GlyRα1, α3L, β, as well as the two chimeras α1α3L and α3Lα1 (with swapped cytoplasmic loops and TM4 domains). The plasmid FU-mEos2-gephyrin for the expression of mEos2 (GenBank FJ707374) tagged rat gephyrin (GenBank X66366, splice variant P1) was derived from an earlier construct [2]. Two bi-cistronic constructs were generated for the expression of non-tagged GlyRα1 or α3 subunits together with soluble EGFP (FU-GlyRα1-IRES-EGFP and FU-GlyRα3-IRES-EGFP; see data in Fig. S3). The above constructs were used for transfection of COS-7 cells and lentivirus production. Lentiviruses were prepared from the FUGW-derived replicons together with the packaging plasmids pCMV-dR8.74 and pMD2.G (Addgene) in HEK-293 cells [3]. Other constructs include FU-HA-hGlyRβ with an HA tag (YPYDVPDYA) inserted after the GlyRβ signal peptide, mRFP-gephyrin [2], and non-tagged versions of GlyRα1 and α3L in mammalian expression vectors.

Cell culture and treatment

African green monkey kidney (COS-7) cells grown in Dulbecco's modified Eagle's medium containing 10% fetal bovine serum (FBS), 2 mM glutamine and antibiotics were transfected with 0.5 µg plasmid DNA per coverslip using FuGENE 6 and used for experiments the following day. Primary cultures of spinal cord neurons were prepared from embryonic Sprague Dawley rats at day 14 (E14) and mRFP-gephyrin knock-in (KI) mice at E13 as described before [2], in accordance with guidelines of the French Ministry of Agriculture and the Direction Départamentale des services véterinaires de Paris (Ecole Normale Supérieure, animalerie des rongeurs, license B 75-05-20). All experiments were done in rat neurons with the exception of the data in Figure 2F (KI mouse cultures). Dissociated spinal cord neurons were plated at a density of 6 x $10^4/cm^2$ on 18 mm coverslips (thickness 130-160 µm), infected with lentivirus at 1-5 days in vitro (DIV) if required, maintained in neurobasal medium with glutamax containing B27, 5 U/ml penicillin and 5 µg/ml streptomycin at 36°C in 5% CO_2, and used for experiments after 14-21 DIV. IL-1β

(Sigma) was applied in culture medium for 15 minutes at a final concentration of 10 ng/ml prior to fixation and immunolabelling, or added to the external solution for electrophysiological recordings.

Immunolabelling

Cells were fixed in 100 mM sodium phosphate buffer pH 7.4 containing 4% PFA and 1% sucrose for 15 min at 36°C, permeabilised in PBS with 0.1% Triton X-100, blocked and labelled with primary antibodies (rabbit anti-GlyRα1, custom made, 1:800; mouse anti-gephyrin mAb7a, Synaptic Systems 147011, 1:1000; mouse anti-GABA$_A$Rβ3, Synaptic Systems 224411, 1:500) and secondary antibodies (Cy3-conjugated goat anti rabbit IgG, Jackson ImmunoResearch, 1:500; Alexa Fluor 647 goat anti mouse, Thermo Fisher Scientific, 1:500) for 1 h in PBS with 3% bovine serum albumin and 0.1% Triton X-100, washed in PBS and mounted on glass slides with Vectashield (Vector Laboratories). For PALM counting of receptor associated mEos2-gephyrin (Fig. 2F), mouse spinal cord neurons were fixed and endogenous GlyRs were labelled with QDs emitting at 605 nm (primary antibody: mouse anti-GlyRα1, Synaptic Systems mAb2b, 1:500; secondary antibody: anti-mouse Fab'-QD$_{605}$, Invitrogen Q11002MP, 1:5000).

Single-particle tracking

The diffusion of Dendra2-tagged GlyRs was measured by single-particle tracking (SPT) using quantum dots (QDs) as described previously [4]. Transfected COS-7 cells or infected spinal cord neurons on glass coverslips were labelled sequentially with Dendra2 antibody (Antibodies-online ABIN361314, 1:10000), biotinylated anti-rabbit F(ab')$_2$ fragments (Jackson, 1:400) and streptavidin coated QDs emitting at 655 nm (Invitrogen Q10121MP, 1:1000), and imaged in MEM imaging medium (phenol red-free minimal essential medium, 20 mM HEPES, 2 mM glutamine, 1 mM sodium pyruvate, 2% FBS, 33 mM glucose) in an open chamber mounted on an inverted microscope (IX71, Olympus) with an oil-immersion objective (63x, NA 1.45) for up to 30 min. QD trajectories were recorded for 500 frames with an exposure time of 75 ms (13 Hz streamed acquisition) using Metamorph software (Molecular Devices). Fluorescence was detected with a xenon lamp and specific filters (Dendra2: excitation 485/20, emission 525/30; QD$_{655}$: ex. 460/60, em. 655/15). Tracking and data analysis were done using homemade software in Matlab (Mathworks). The centre of QD signals was determined by Gaussian fitting. Only trajectories of at least 15 consecutive frames were considered for quantification. Diffusion coefficients D were calculated by fitting the first five points of the mean square displacement (MSD) plot against time.

Supplemental figures and tables

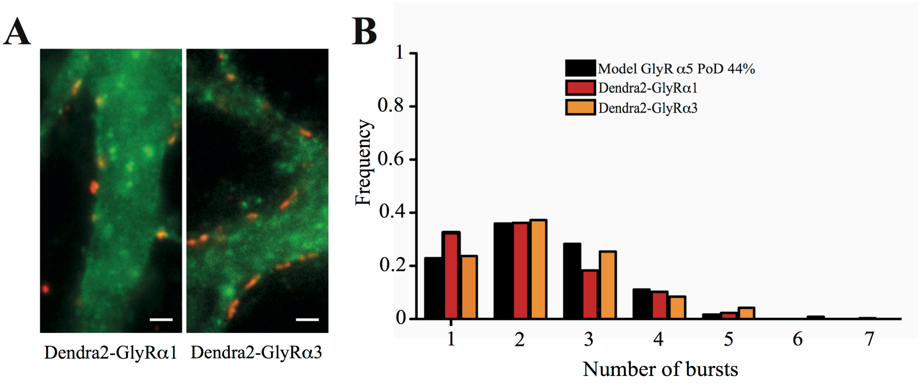

Figure S1. GlyRs form homopentameric complexes in hippocampal neurons.

(A) Conventional fluorescence microscopy of hippocampal neurons at DIV14 expressing Dendra2-GlyRα1 or α3 (green) and stained for gephyrin (red). Scale bar: 1 µm. Hippocampal neurons lack endogenous GlyRβ subunits. Consequently, the fluorescence associated with Dendra2-tagged GlyRs is diffusely distributed on the dendrites and does not co-localise with the scaffold protein gephyrin at inhibitory synapses.

(B) The burst frequencies of Dendra2-tagged GlyRα1 (red bars) or GlyRα3 (orange) in extrasynaptic regions of infected hippocampal neurons are comparable with those expected from a homopentameric α_5 receptor stoichiometry and a probability of detection p_{det} = 0.44 of the Dendra2 fluorophores (black bars; counts and fitting results are given in Table S1). This is in line with the absence of endogenous GlyRβ subunits in these neurons [5].

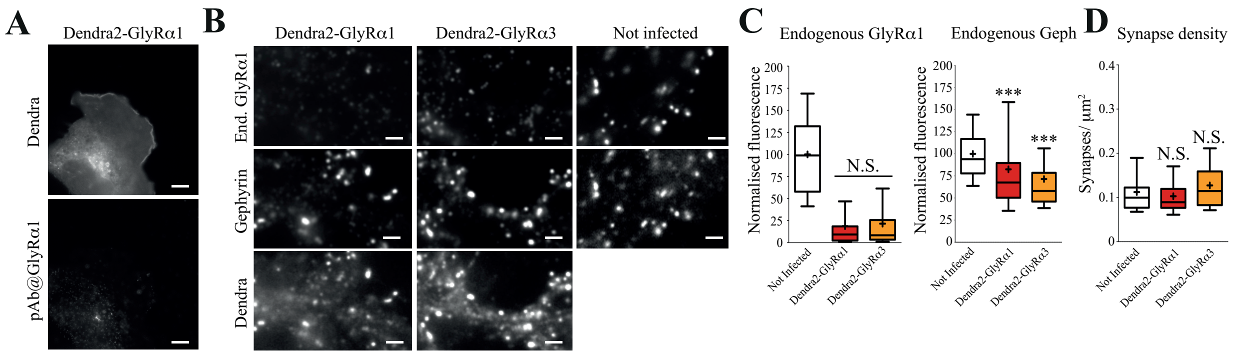

Figure S2. Endogenous GlyRα1 replacement by Dendra2-tagged subunits at synapses.

(A) Conventional fluorescence microscopy of COS-7 cells transfected with Dendra2-GlyRα1 (top panel) and labelled with an antibody against the N-terminus of GlyRα1 (bottom). The antibody fails to recognise the Dendra2-tagged version of GlyRα1, making it a useful tool to discriminate between the signals associated with endogenous versus recombinant GlyRs. Scale bar: 5 µm.

(B) Fluorescence images of spinal cord neurons (DIV14) stained for endogenous GlyRα1 (top panels) and gephyrin (middle). In infected neurons (Dendra2 signals, bottom panels) the endogenous GlyRα1 levels are substantially decreased compared to non-infected neurons. Scale bar: 3 µm.

(C) Quantification of the background-corrected integrated fluorescence intensity of endogenous GlyRα1 in dissociated spinal cord cultures. Overexpression of recombinant Dendra2-GlyRα1 and α3 reduces the endogenous receptor levels by about 80% ($n \geq 46$ cells per condition from 3 independent experiments; MW $p < 0.0001$ versus non-infected neurons; α1 versus α3 $p = 0.8$). Overexpression of recombinant GlyRs in these neurons decreased endogenous gephyrin levels at synapses compared to non-infected controls (MW $p = 0.0002$ for α1 and $p < 0.0001$ for α3).

(D) The density of inhibitory synapses was not different between infected and non-infected neurons, as judged by the number of gephyrin positive puncta.

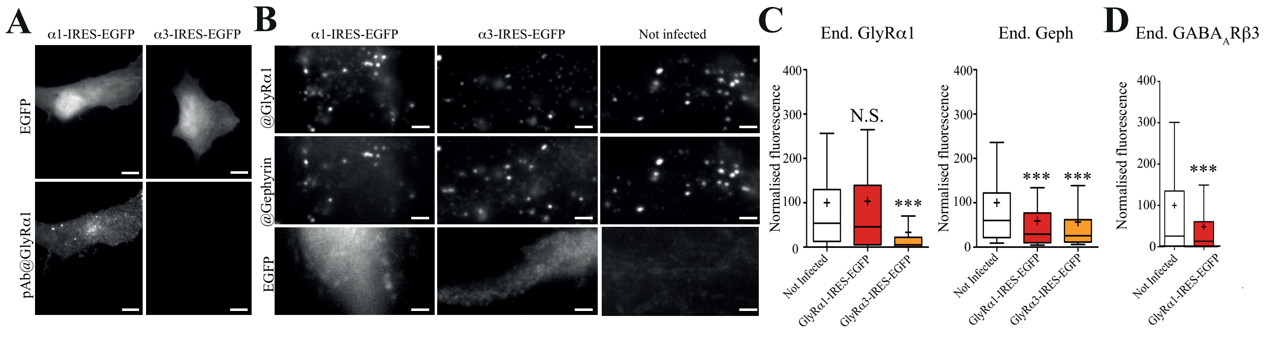

Figure S3. Effects of lentiviral expression of non-tagged GlyRs on inhibitory synaptic components.

(A) Conventional fluorescence microscopy of COS-7 cells transfected with FU-GlyRα1-IRES-EGFP and FU-GlyRα3-IRES-EGFP (top panels) and labelled with an antibody against GlyRα1 (bottom panels). Antibody labelling is specific for the GlyRα1 subunit (bottom left) and shows no cross-reactivity with GlyRα3 (bottom right). These constructs therefore enable the quantification of total GlyRα1 levels in α1 and α3 expressing cells and non-infected controls (shown in B). Scale bar: 5 µm.

(B) Fluorescence images of dissociated spinal cord neurons (DIV14) stained for endogenous GlyRα1 (top panels) and gephyrin (middle). In neurons infected with GlyRα1-IRES-EGFP (left) the total levels of GlyRα1 do not differ from those of non-infected controls (right). The expression of GlyRα3, instead, substantially decreases GlyRα1 levels compared to non-infected neurons, confirming that lentiviral expression of GlyRα subunits largely replaces endogenous GlyRα1 at synapses. Scale bar: 3 µm.

(C) Quantification of the integrated fluorescence intensity of total GlyRα1 clusters at spinal cord synapses. The lentiviral expression of non-tagged GlyRα1 did not alter the total levels of α1-containing GlyRs compared to non-infected cells ($n > 400$ clusters, MW $p = 0.16$). Overexpression of GlyRα3 reduced the levels of endogenous GlyRα1 by around 70% (MW $p < 0.0001$), confirming that lentiviral expression of non-tagged recombinant GlyRs replaces the endogenous GlyRα1 in infected neurons (compare Fig. S2C). Synaptic gephyrin fluorescence was decreased in lentivirus infected neurons (see also Fig. S2C).

(D) Quantification of GABA$_A$Rβ3 integrated fluorescence intensity in GlyRα1-positive clusters. The expression of recombinant GlyRα1 reduced by 50% the levels of GABA$_A$Rβ3 at synapses with respect to non-infected cells ($n > 350$ clusters, MW $p < 0.0001$).

Table S1. Single fluorophore analysis of GlyR stoichiometry.

COS-7 cells, rat spinal cord and hippocampal neurons expressing Dendra2-tagged GlyR subunits (generally from ≥ 6 cells and 3 independent experiments) were used for quantitative PALM. Bursts of detections (raw counts) were counted in the time traces of isolated clusters of detections as described in the Methods section (excluding synaptic clusters). The known homopentameric stoichiometry of GlyRα subunits was used to fit the raw counts with a binomial distribution of n = 5, yielding the probability of detection p_{det} of the Dendra2 fluorophore. The goodness of fit was assessed using a chi-square test. The average $p_{det} = 0.44$ was then applied to the normalised burst frequencies in order to identify the subunit stoichiometries (n = 2 to 5) associated with the smallest residuals (shown in bold).

Table S2. Single fluorophore counting of gephyrin complexes.

Using PALM imaging, we counted the number of bursts (raw counts) in the time traces of isolated clusters of detections in COS-7 cells and in the non-synaptic regions of mouse spinal cord neurons expressing mEos2-gephyrin (from ≥ 6 cells and 3 independent experiments). Counts were fitted with a binomial distribution of n = 3 or 6. The trimeric stoichiometry of gephyrin was validated using a chi-square test and by taking into account the associated probability of detection p_{det} of mEos2.

References

1 Lois, C., Hong, E. J., Pease, S., Brown, E. J. & Baltimore, D. Germline transmission and tissue-specific expression of transgenes delivered by lentiviral vectors. *Science* **295**, 868-872 (2002).

2 Specht, C. G. *et al.* Quantitative nanoscopy of inhibitory synapses: counting gephyrin molecules and receptor binding sites. *Neuron* **79**, 308-321 (2013).

3 Waites, C. L. *et al.* Synaptic SAP97 isoforms regulate AMPA receptor dynamics and access to presynaptic glutamate. *J Neurosci* **29**, 4332-4345 (2009).

4 Specht, C. G. *et al.* Regulation of glycine receptor diffusion properties and gephyrin interactions by protein kinase C. *EMBO J* **30**, 3842-3853 (2011).

5 Weltzien, F., Puller, C., O'Sullivan, G. A., Paarmann, I. & Betz, H. Distribution of the glycine receptor beta-subunit in the mouse CNS as revealed by a novel monoclonal antibody. *J Comp Neurol* **520**, 3962-3981 (2012).

Table S1

Cell type	Construct	# cells	Raw counts 1	2	3	4	5	6	7	sum	p_{det} n=5	chi^2
COS - 7	Dendra2-GlyRα1	9	540	777	554	195	52	18	4	2140	0.42	10.3
COS - 7	Dendra2-GlyRα3	6	331	636	500	211	36	17	6	1737	0.458	3.9
COS - 7	Dendra2-GlyRα1+ HA-GlyRβ	8	708	697	294	84	21	5	1	1810		
COS - 7	Dendra2-GlyRα3+ HA-GlyRβ	7	781	565	267	63	15	1	0	1692		
COS - 7	Dendra2-GlyRβ + GlyRα3	6	238	91	22	10	1	1	0	363		
COS - 7	Dendra2-GlyRβ + GlyRα1	10	372	167	42	13	4	1	1	600		
spinal cord neurons	Dendra2-GlyRα1	6	217	187	89	34	5	2	0	534		
spinal cord neurons	Dendra2-GlyRα3	6	220	126	52	8	3	0	0	409		
spinal cord neurons	Dendra2-GlyRβ	7	256	100	26	3	3	0	0	388		
hippocampal neurons	Dendra2-GlyRα1	3	98	109	55	31	7	0	1	301		
hippocampal neurons	Dendra2-GlyRα3	2	28	44	30	10	5	1	0	118		

Cell type	Construct	Frequencies 1	2	3	4	5	6	7	sum (1-7)	p_{det}	squared residuals n=2	n=3	n=4	n=5
COS - 7	Dendra2-GlyRα1	0.252	0.363	0.259	0.091	0.024	0.008	0.002	1	0.44	0.299	0.097	0.015	**0.002**
COS - 7	Dendra2-GlyRα3	0.191	0.366	0.288	0.121	0.021	0.010	0.003	1	0.44	0.383	0.147	0.037	**0.002**
COS - 7	Dendra2-GlyRα1+ HA-GlyRβ	0.391	0.385	0.162	0.046	0.012	0.003	0.001	1	0.44	0.146	**0.018**	**0.005**	0.046
COS - 7	Dendra2-GlyRα3+ HA-GlyRβ	0.462	0.334	0.158	0.037	0.009	0.001	0	1	0.44	0.095	**0.010**	0.022	0.076
COS - 7	Dendra2-GlyRβ + GlyRα3	0.656	0.251	0.061	0.028	0.003	0.003	0	1	0.44	**0.009**	0.047	0.144	0.250
COS - 7	Dendra2-GlyRβ + GlyRα1	0.620	0.278	0.070	0.022	0.007	0.002	0.002	1	0.44	**0.015**	0.029	0.113	0.213
spinal cord neurons	Dendra2-GlyRα1	0.406	0.350	0.167	0.064	0.009	0.004	0	1	0.44	0.134	**0.019**	**0.010**	0.047
spinal cord neurons	Dendra2-GlyRα3	0.538	0.308	0.127	0.020	0.007	0	0	1	0.44	0.050	**0.010**	0.055	0.131
spinal cord neurons	Dendra2-GlyRβ	0.660	0.258	0.067	0.008	0.008	0	0	1	0.44	**0.009**	0.045	0.144	0.253
hippocampal neurons	Dendra2-GlyRα1	0.326	0.362	0.183	0.103	0.023	0	0.003	1	0.44	0.205	0.050	**0.007**	**0.019**
hippocampal neurons	Dendra2-GlyRα3	0.237	0.373	0.254	0.085	0.042	0.008	0	1	0.44	0.313	0.102	0.018	**0.002**

Table S2

Cell type	Construct	# cells	Raw counts												
			0	1	2	3	4	5	6	7	sum (1-7)	p_{det} n=3	chi^2	p_{det} n=6	chi^2
COS - 7	Eos2-Gephyrin	6	-	640	571	296	99	15	6	1	1628	0.524	25.9	0.277	7.7
spinal cord neurons (KI)	Eos2-Gephyrin	14	-	994	725	330	116	30	12	4	2211	0.477	40.6		
spinal cord neurons (KI)	QD-GlyRα1 + Eos2-Gephyrin	10	22	14	10	4	0	0	0	0	28	0.434	0.06		

Cell type	Construct	Frequencies							
		1	2	3	4	5	6	7	sum (1-7)
COS - 7	Eos2-Gephyrin	0.393	0.351	0.182	0.061	0.009	0.004	0.001	1
spinal cord neurons (KI)	Eos2-Gephyrin	0.450	0.328	0.149	0.052	0.014	0.005	0.002	1
spinal cord neurons (KI)	QD-GlyRα1 + Eos2-Gephyrin	0.500	0.357	0.143	0.000	0.000	0.000	0.000	1
